# The influence of primary and subsequent limb amputation on the overall rate of limb amputation in Saskatchewan, Canada, 2006–2019: a population-based study

**DOI:** 10.1186/s12893-021-01381-2

**Published:** 2021-10-30

**Authors:** Samuel Kwaku Essien, A. Gary Linassi, Colin Farnan, Kassondra Collins, Audrey Zucker-Levin

**Affiliations:** 1 School of Rehabilitation Science, University of Saskatchewan, Health Science Building, E-Wing, Suite 3400, 3rd Floor, 104 Clinic Place, Saskatoon, SK S7N 2Z4 Canada; 2 Department of Physical Medicine and Rehabilitation, University of Saskatchewan, Saskatoon, SK S7K 0M7 Canada; 3 Patient-Oriented Team (PORT), 104 Clinic Place, Saskatoon, SK S7N 2Z4 Canada

**Keywords:** Epidemiology, Amputation, Trends, Incidence

## Abstract

**Background:**

Understanding trends in limb amputation (LA) can provide insight into the prevention and optimization of health care delivery. We examine the influence of primary (first report) and subsequent (multiple reports) limb amputation on the overall (all reports) rate of limb amputation in Saskatchewan considering amputation level.

**Methods:**

Hospital discharged data associated with LA from 2006 to 2019 and population estimates in Saskatchewan were used. LA cases were grouped based on overall, primary, and subsequent LA and further divided by level into major (through/above the ankle/wrist) and minor (below the ankle/wrist). Incidence rates were calculated using LA cases as the numerator and resident population as the denominator. Joinpoint and negative binomial were used to analyze the trends. In addition, the top three amputation predisposing factors (APF) were described by LA groups.

**Results:**

The rate of overall LA and primary LA remained stable (AAPC − 0.9 [95% CI − 3.9 to 2.3]) and (AAPC −1.9 [95% CI −4.2 to 0.4]) respectively, while the rate of subsequent LA increased 3.2% (AAPC 3.2 [95% CI 3.1 to 9.9]) over the 14-year study period. The rate of overall major LA declined 4.6% (AAPC − 4.6 [95% CI −7.3 to −1.7]) and was largely driven by the 5.9% decline in the rate of primary major LA (AAPC − 5.9 [95% CI − 11.3 to –0.2]). Subsequent major LA remained stable over the study period (AAPC −0.4 [95% CI − 6.8 to 6.5]). In contrast, the overall rate of minor LA increased 2.0% (AAPC 2.0 [95% CI 1.0 to 2.9]) over the study period which was largely driven by a 9.6% increase in the rate of subsequent minor LA (AAPC 9.6 [95% CI 4.9 to 14.4]). Primary minor LA rates remained stable over the study period (AAPC 0.6 [95% CI − 0.2 to 1.5]). The study cohorts were 1.3-fold greater risk of minor LA than major LA. Diabetes mellitus (DM) was the leading APF representing 72.8% of the cohort followed by peripheral vascular disease (PVD) and trauma with 17.1 and 10.1% respectively. Most (86.7%) of subsequent LA were performed on people with DM.

**Conclusions:**

Overall LA rates remained stable over the study period with declining rates of major LA countered by rising rates of minor LA. Minor LA exceeded major LA with the largest rate increase identified in subsequent minor LA. Diabetes was the greatest APF for all LA groups. This rising rate of more frequent and repeated minor LA may reflect changing intervention strategies implemented to maintain limb function. The importance of long-term surveillance to understand rates of major and minor LA considering primary and subsequent intervention is an important step to evaluate and initiate prevention and limb loss management programs.

## Background

Limb amputation (LA) is most frequently performed to avert deterioration of health in patients with chronically infected wounds due to peripheral arterial disease (PAD) associated with diabetes mellitus (DM) [1–3]. Other causes of LA include other vascular diseases, trauma, infection, malignancy, and congenital anomalies respectively [4–8]. Independent of cause, major LA (through/above the ankle/wrist joint) is associated with poor quality of life [9], excessive burden and stress on persons, families, and the health care systems [10], and high mortality [11–13]. In contrast, minor LA (below the ankle/wrist joint), preserves function and may reduce the need for major LEA [14]. Subsequent amputation (revision or contralateral amputation) may be necessary after major or minor LA to mitigate disease progression, improve prosthetic fit, or decrease pain. Revision rates up to 40.9% [1] have been recently reported for people with DM and remnant infection. This is of critical concern as Saskatchewan has observed a 45% increase in the prevalence of DM in the last decade [15] and experienced the highest DM-related hospital admission rates among Canadian provinces between 2013 and 2014 [16].

Hence, understanding the proportion and the patterns of primary (first report) and subsequent LA (multiple reports) can act as a proxy to evaluate therapeutic and surgical interventions directed at limb salvage for LA due to both trauma and disease [17, 18].

The multidisciplinary patient-oriented research team (PORT) comprised of people with amputation, caregivers, researchers, educators, and health care providers was created to focus on amputee health and well-being in Saskatchewan, Canada. The PORT identified the need to understand the specific epidemiology of LA in Saskatchewan with the ultimate goal to determine how Saskatchewan LA rates compare to other Canadian provinces and globally. Currently, two recent reports provide data on LA rates in Canada. Hussain et al. describe trends in the rate of LA due to DM and/or PAD in Ontario, Canada for 2005–2016 [19]. While Imam et al. captured national and provincial per capita demographics of LA spanning 2006 to 2011 [20]. Although valuable, Imam identifies the need to interpret the results with caution as the incidence rates were calculated on unequal time-interval of cases (fiscal years) and populations (years). Thus, the annual or fiscal year rate of LA for each province may not be accurately reflected [20].

Determining the extent to which LA affects population groups in Saskatchewan is crucial for informing intervention strategies. Our primary objective was to describe the LA rate in Saskatchewan from 2006 to 2019 considering three groups: overall LA cases (all recorded amputations), primary LA cases (first report), and subsequent LA cases (multiple reports) with further exploration of rates of major and minor amputations within each group.

## Methods

### Study type/design

We performed a descriptive cross-sectional study to examine trends in the rates of overall, primary, and subsequent LA while considering amputation level (major or minor) in Saskatchewan, Canada including years 2006–2019.

### Study population

We identified residents of Saskatchewan of all ages who underwent LA, independent of cause, between January 1, 2006, and December 31, 2019.

### Setting/data sources

The data used in this study include Saskatchewan’s comprehensive administrative health databases housed at eHealth Saskatchewan accessible to the Saskatchewan Health Quality Council. Hospital data, coded by trained in-house administrative staff using the International Classification of Diseases, 10th revision, Canadian Version (i.e., ICD-10-CA) and companion Canadian Classification of Health Interventions (CCI), is contained in the *Discharge Abstract Database (DAD)*. The DAD captures up to 25 diagnoses and up to 20 interventions per hospitalization and is anonymously linked, by unique personal health insurance numbers, to the *Person Health Registration System* that captures demographic characteristics and dates of coverage by the provincial health insurance plan. The accuracy and completeness of Saskatchewan's administrative databases have made them popular sources for numerous studies of population health and health services utilization [21–24].

Retrospective data from Saskatchewan on all persons discharged from a Saskatchewan hospital, with recorded CCI codes for amputation (1SN93, 1SQ93, 1TA93, 1TK93, 1TM93, 1TV93, 1VA93, 1VC93, 1VG93, 1VQ93, 1UB93, 1UE93, 1UF93, 1UG93, 1UH93, 1UI93, 1UJ93, 1UK93, 1UM93, 1WA93, 1WE93, 1WI93, 1WJ93, 1WK93, 1WL93, 1WM93, 1WN93) [25] in any of the 20 intervention fields, spanning the period January 1, 2006, to December 31, 2019, were extracted from the DAD. The start point of 2006 was selected because it corresponds to the implementation of ICD-10-CA/CCI classifications allowing for comparison to other provinces across Canada [26–28]. The annual Saskatchewan resident population from 2006 to 2019, obtained from the Saskatchewan Bureau of Statistics [29], was used as denominators for LA rate estimates. The extracted data were categorized into three groups: (1) overall amputation cases (includes all amputation cases) (2) primary amputation cases (the first report of amputation in an individual) and (3) subsequent amputations (any report of an additional amputation, revision or contralateral, in an individual identified in the primary amputation group). All groups were further divided into major LA (through/above the ankle/wrist joint) and minor amputation (below the ankle/wrist joint) [30]. Data on the study subject’s demographic factors including age, sex, and admission date were also extracted. Identifying the direct cause of LA in this dataset was not possible and precluded any causal inference to be drawn. As a surrogate, the top three co-morbidities present at the time of LA, identified as amputation predisposing factors (APF), were tabulated based on ICD-10-CA diagnostic codes including diabetes (e.g., E10-E14), vascular diseases (e.g., I70, I72-I78, and I80-I99) and trauma (e.g., S480-S481, S680-S684, S980-S984, and T050-T059). This study followed the globally accepted STROBE recommendations for reporting observational studies [31] for the enhancement of the study quality and comparability to other studies. Ethical approval was obtained from the University of Saskatchewan Biomedical Ethics Board (U of S # Bio 1590).

### Statistical analysis

For the primary analysis, we examined trends of annual rates and the top three APF of three LA groups: overall, primary, and subsequent amputation. In addition, trends and annual rates of major and minor LA within each group were explored. Our secondary analyses examined the differential impact of minor and major LA on the overall LA rate. Firstly, to understand population demographics, the proportions comparing the top three APF in the study population were explored and reported. Also, the yearly amputation rates were calculated by dividing the total LA cases for each year by the annual Saskatchewan resident population expressed as per 100,000 populations. T-tests were used to determine differences in rates between primary and subsequent LA and major and minor LA with a significance set p < 0.05.

Joinpoint regression analysis [32] was employed to detect significant changes in LA rate trends over the fourteen years (2006–2019). The grid search method inherent in the Joinpoint program was used to fit the model and the permutation test based on Monte Carlo resampling was employed to select the number of breakpoints [32, 33]. The model process begins with a selected minimum number of breakpoints and statistically tests whether one or more breakpoints are significantly different from zero [32, 33]. The annual percent change (APC) and the average annual percent change (AAPC) were reported from the models with a 95% confidence interval (Cl). In the event where no breakpoint is found, the estimated value of APC and AAPC are equal. The Joinpoint regression program software, version 4.8.0.1 was used in all Joinpoint-related analyses [32]. The mathematical algorithms upon which the software executes/operates are reported elsewhere [33].

Finally, a negative binomial regression was performed to test whether there were statistically significant differences in time trends in major vs. minor LA rates [34]. These differences were examined by first fitting an unadjusted model between overall LA rate and levels of LA and then the adjusted models were fitted by including the year of LA as a continuous variable and an interaction term. The interaction term was created by multiplying the year of LA by levels of LA. The relative rate (RR) and 95% CI were estimated and reported from the models, with a significance set at p < 0.05. A statistically significant interaction term was an indication that the rate of change of overall LA was differentially impacted by the levels of LA.

## Results

### Demographic and overall trends

From 2006 to 2019 there were 5868 LA cases in Saskatchewan. Of these, 4239 (72.2%) were primary and 1629 (27.8%) were subsequent LA cases. More than half, 62.4% (3661) of all cases from 2006 to 2019 were minor and 37.6% (2207) were major. The average (SD) age was 60.4 ± 19.1 years and 71% were males.

The three top APF, representing 75% (4421) of all LA procedures were compared among overall, primary, and subsequent LA cohorts (Table 1). Diabetes mellitus (DM) was the leading APF representing 72.8% of the cohort followed by peripheral vascular disease (PVD) and trauma with 17.1 and 10.1% respectively. Likewise, DM was the leading APF present in 86.7% of subsequent LA cases followed by PVD and trauma with 12.3 and 1% respectively. The 25% of cases not included in this analysis each had low frequency (less than 10% of the overall cohort) with infection identified as the most frequent, followed by cancer, and congenital.

**Table 1:** Characteristics of most frequent amputation predisposing factors by the level of amputation

Amputation predisposing factor (APF)	Overall N (%)	Primary N (%)	Subsequent N (%)
Diabetes	3220 (72.8)	2043 (66.7)	1177 (86.7)
Peripheral vascular disease	754 (17.1)	586 (19.1)	168 (12.3)
Trauma	447 (10.1)	434 (14.2)	13 (1.0)

### Overall, primary and subsequent amputation rates

**Fig. 1:**
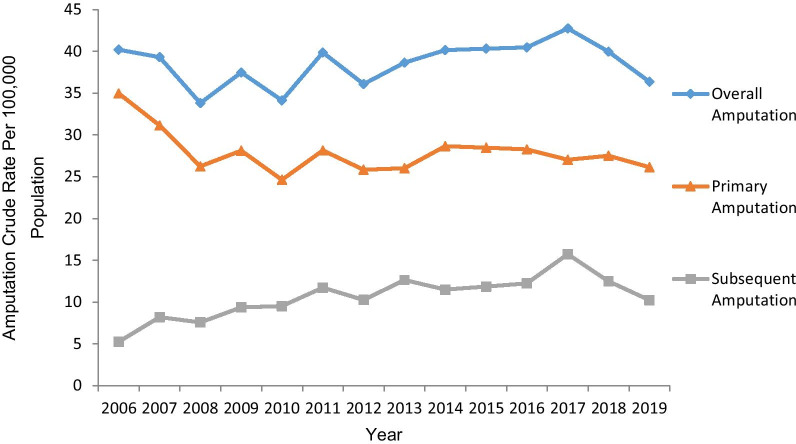
Crude rates of overall amputation, primary amputation, and subsequent amputation 2006–2019

Crude rates of overall, primary, and subsequent LA in Saskatchewan from 2006 to 2019 are presented in Fig. 1. The overall LA rate was 38.55 ± 2.6 per 100,000 population; the primary LA rate, 27.94 ± 2.6 per 100,000 population, significantly exceeded the subsequent LA rate, 10.6 ± 2.6 per 100,000 population over the entire study period (p < 0.001).

The Joinpoint analysis (Table 2) revealed both overall LA and primary LA rates remained stable (AAPC -0.9 [95% CI − 3.9  to 2.3]) and (AAPC − 1.9 [95% CI − 4.2 to 0.4]) respectively, while the rate of subsequent LA increased 3.2% (AAPC 3.2 [95% CI 3.1 to 9.9]) (p < 0.05) over the 14 year study period. Rates fluctuated over the study period including a 2.1% increase in the rate of overall LA from 2008–2017 (APC 2.1 [95% CI 0.3 to 4.0]) (p < 0.05) countered by two insignificant periods of decline (2006–2008 and 2017–2019). The fluctuation in overall LA nearly corresponds with the 7.5% (APC 7.5 [95% CI 4.3 to 10.7]) (p < 0.05) increase in the rate of subsequent LA from 2006–2017 followed by an insignificant period of decline from 2017 to 2019. No significant fluctuations were identified in the rate of primary LA.

**Table 2:** Annual percent change in amputation rates, 2006–2019

Amputation Rate	Breakpoints	APC (95%CI)	Full Range	AAPC 95% CI)
Overall	2006–2008 2008–2017 2017–2019	− 7.7 (− 22.0 to 9.2) 2.1* (0.3 to 4.0) − 6.8 (−21.2 to 10.3)	− 0.9	(− 3.9 to 2.3)
Primary	2006–2008 2008–2019	− 13.0 (− 26.3 to 2.8) 0.3 (− 0.9 to 1.4)	− 1.9	(− 4.2 to 0.4)
Subsequent	2006–2017 2017–2019	7.5* (4.3 to 10.7) − 17.5 (−46.9 to 28.3)	3.2*	(3.1 to 9.9)
Overall major	2006–2010 2010–2017 2017–2019	− 11.1* (−16.1 to − 5.9) 4.6* (1.4 to 7.8) − 20.0* (−33.3 to − 4.2)	− 4.6*	(− 7.3 to − 1.7)
Overall minor	2006–2019	2.0* (1.0 to 2.9)	2.0*	(1.0 to 2.9)
Primary major	2006–2010 2010–2016 2016–2019	− 15.1* (−25.3 to − 3.6) 3.7 (− 5.3 to 13.4) − 11.0 (− 27.3 to 8.8)	− 5.9*	(− 11.3 to − 0.2)
Primary minor	2006–2019	0.6 (− 0.2 to 1.5)	0.6	(− 0.2 to 1.5)
Subsequent major	2006–2017 2017–2019	5.4* (2.1 to 8.8) − 26.8 (− 54.3 to 17.2)	− 0.4	(− 6.8 to 6.5)
Subsequent minor	2006–2010 2010–2019	31.0* (14.4 to 50.2) 1.2 (− 2.7 to 5.3)	9.6*	(4.9 to 14.4)

*APC* Annual Percent Change, *AAPC* Average Annual Percent Change, *Cl* Confidence Interval

*Indicates a statistically significant breakpoint

**Fig. 2:**
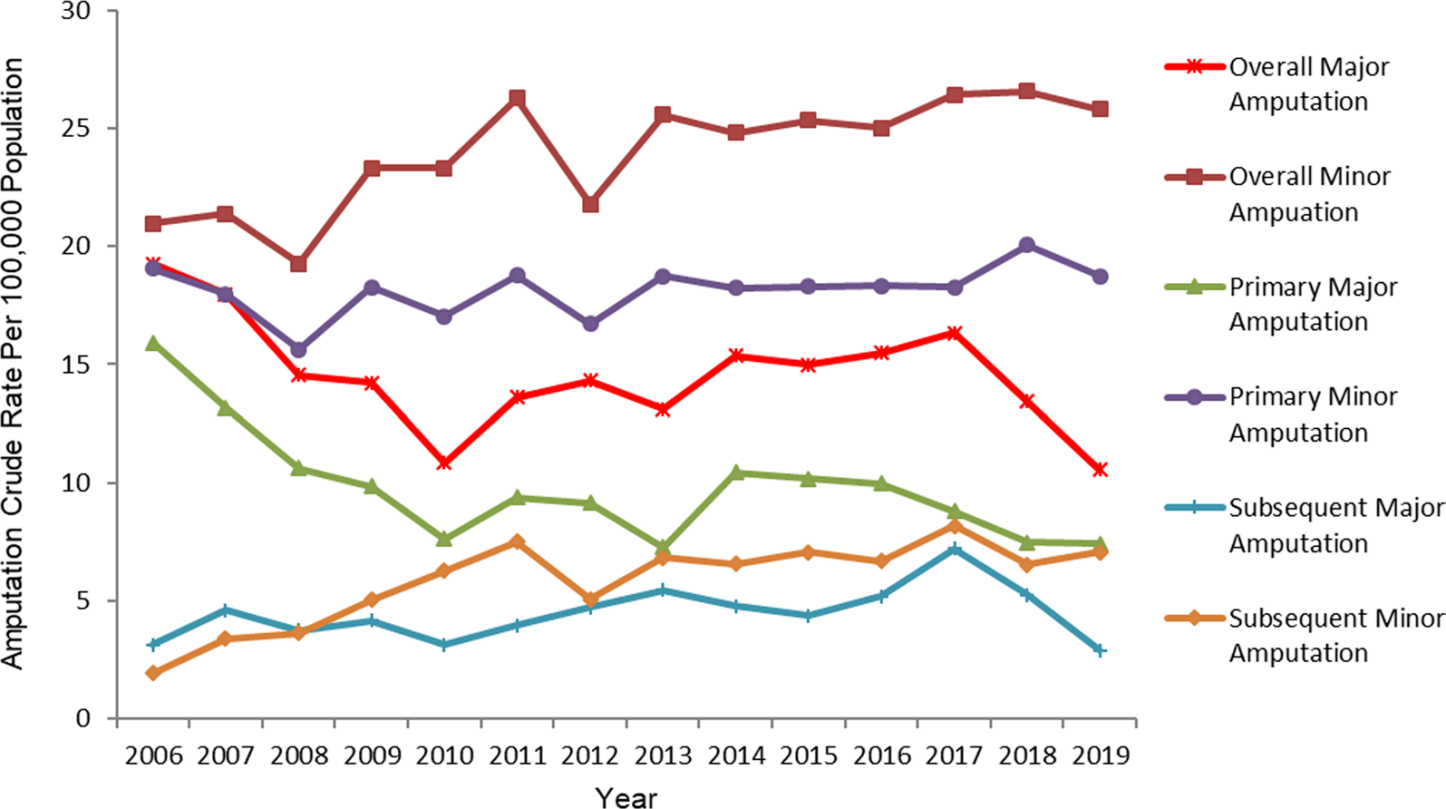
Crude rates of amputations in Saskatchewan stratified by calendar year, group, and level of amputation

Crude rates of major and minor LA stratified by calendar year and group are presented in Fig. 2. The rates of minor LA exceeded major LA in all groups during the study period: overall (minor 23.9 ± 2.34, major 14.57 ± 2.28; p < 0.001), primary (minor 18.14 ± 1.08***,*** major 9.8 ± 2.39; p < 0.001), and subsequent (minor 5.84 ± 1.79, major 4.47 ± 1.14; p < 0.001).

Joinpoint analysis (Table 2) revealed both overall major LA and primary major LA significantly decreased 4.6% (AAPC − 4.6 [95% CI − 7.3 to − 1.7]) (p < 0.05), and 5.9% (AAPC − 5.9 [95% CI − 11.3 to − 0.2]) (p < 0.05) respectively while subsequent major LA remained stable (AAPC − 0.4 [95% CI − 6.8 to 6.5]) over the study period. Fluctuations were identified in the rates of major LA among all groups. A 4.6% increase in the rate of overall major LA from 2010–2017 (APC 4.6 [95% CI 1.4 to 7.8]) (*p* < 0.05) was countered by two periods of decrease; 11.1% from 2006–2010 (APC − 11.1 [95% CI − 16.1 to − 5.9]) (*p* < 0.05) and 20% from 2017 to 2019 (APC − 20.0 [95% CI − 33.3 to − 4.2]) (p < 0.05). This nearly corresponded with the fluctuations in rate of primary major LA with an insignificant increase from 2010–2016 (APC 3.7 [95% CI − 5.3 to 13.4]) countered by a decrease of 15.1% from 2006–2010 (APC − 15.1 [95% CI − 25.3 to − 3.6]) (p < 0.05) and an insignificant decrease from 2016 to 2019 (APC -11.0 [95% CI − 27.3 to 8.8]). Finally, subsequent major LA increased 5.4% from 2006 to 2017 (APC 5.4 [95% CI 2.1 to 8.8]) (p < 0.05) and insignificantly decreased from 2017–2019 (APC − 26.8 [95% CI −54.3 to 17.2]). The significant decrease in overall major LA can be attributed to the significant decrease in primary major LA.

Joinpoint analysis further revealed both overall minor LA and subsequent minor LA significantly increased 2.0% (AAPC 2.0 [95% CI 1.0 to 2.9]) (p < 0.05), and 9.6% (AAPC 9.6 [95% CI 4.9 to 14.4]) (p < 0.05) respectively while primary minor LA remained stable (AAPC 0.6 [95% CI − 0.2 to 1.5]) over the study period. The only group to experience rate fluctuation over the study period was that of subsequent minor LA with a rise of 31.0% from 2006 to 2010 (APC 31.0 [95% CI 14.4 to 50.2]) (p < 0.05) further bolstered by an insignificant increase from 2010 to 2019 (APC 1.2 [95% CI − 2.7 to 5.3]). The significant rise in overall minor LA can be attributed to the significant rise in subsequent minor LA.

The findings of the Joinpoint analyses are further supported by the negative binomial analysis (Table 3) which revealed the study cohorts were 1.65 times more likely to have minor LA compared to major LA, as evidenced by the unadjusted model (RR = 1.65, 95% CI 1.53–1.77). Accounting for the year of LA and level of amputation-by-year of LA interaction, the relative rate decreased to 1.26 (RR = 1.26, 95% CI 1.08–1.46). This clearly shows that the study cohort was 1.3-fold greater risk of minor LA than major LA and that minor LA contributed substantially to the increased rate of overall LA in the study population. Additionally, the level of LA by year of LA interaction was statistically significant (p < 0.001), suggesting that the rate of change of overall LA was differentially impacted by the levels of LA.

**Table 3:** Unadjusted and adjusted relative rates for amputations among population groups

Variables	Model							
	Unadjusted				Adjusted			
Amputation level	Coeff	RR	95% CI	P-value	Coeff	RR	95% CI	P-value
Major		1.00				1.00		
Minor	0.498	1.65	(1.53–1.77)	< 0.001	0.228	1.26	(1.08–1.46)	0.003
Year					− 0.053			< 0.001
Amputation level and Year Int*					0.036			< 0.001

*RR* Relative Rate, *Cl* Confidence Interval, Int* Interaction,  *Coeff* Coefficient

## Discussion

This is the first study to have explicitly explored the epidemiology and recent trends of LA in Saskatchewan over 14 years. We chose to study all causes and levels of LA to identify the true scope of the social and economic burden placed on the health care system, family, and caregivers [24]. This was important as other Canadian researchers limited their studies based on diagnosis (e.g., diabetes, trauma) level (major, minor), or extremity (upper, lower) [19, 20, 35–38]. To add some understanding of the cohort demographics, we included the three most common amputation predisposing factors (APF) and identified DM as the leading cause of limb loss followed by PVD and trauma among all groups. This distribution was not surprising and is consistent with other epidemiologic studies of LA. Further, our use of yearly comparable cases and denominators makes the estimated rates more reflective of the annual rates of LA. The data showed that over fourteen years (2006–2019), there were 5868 amputations performed in Saskatchewan (annualized rate of 38.55 ± 2.6 per 100,000 population), with 72.2% being a primary LA and 27.8% subsequent LA. The rate of overall LA and the rate of primary LA remained stable over 2006–2019 while the subsequent LA rate increased 3.2%. Minor LA is significantly more common than major LA with a 4.6% decrease in the rate of major LA and a 2% increase in the rate of minor LA observed during the study period. We found primary major LA rates decreased 5.9% while primary minor LA rates remained stable and subsequent minor amputation rates increased 9.6% while subsequent major LA rates remained stable over the study period.

Comparison of our findings to other reports reveals a higher annualized LA rate of 38.55 ± 2.6 per 100,000 in the Saskatchewan population than that reported by both Imam et al. (Saskatchewan: 28.3 per 100,000) [20] and Hussain et al. (Ontario: 10 per 100,000) [19]. This disparity may be due to our inclusion of all amputations performed in the province while Imam and Hussein limited their studies to lower extremity amputation (LEA), with Hussein further limiting inclusion to LEA caused by DM and/or PAD in individuals 40 years of age and older.

Trends in the LA rate also differed slightly among Canadian reports [19, 20]. We found a stable rate of overall LA between 2006 and 2019 while Hussain et al. found an increase in the rate of LEA in Ontario, Canada between 2005 and 2016 [19]. This difference may be explained by the study population differences and different time periods as we identified a 2.1% rise in the overall LA rate between 2008 and 2017. Further exploration of time periods revealed the 4.6% decline in overall major LA and the 5.9% decline in primary major LA were largely driven by the years 2006–2010. Unfortunately, the decline in primary major LA was contrasted by a rise in the rate of subsequent major (5.4%) and subsequent minor (31%) LA encompassing the same time period. The sharp rise in primary and overall LA rates, especially observed in 2012, from 25.84 and 36.08 per 100,000 to 36.08 and 40.16 per 100,000 in 2014 respectively could be due to differing proportions of major causes (DM associated PAD, other vascular diseases, trauma, infection, cancer, and congenital anomalies) [4–8, 20] of LA during these time intervals. For example, from 2013–2014 Canadian Institute for Health Information (CIHI) data revealed that Saskatchewan DM hospital admissions rate was the highest (186.5 admissions per 100,000 population) when compared with other Canadian provinces [16].

Our finding of a 2% increase in the overall rate of minor amputation from 2006–2019 is supported by other studies with diverse populations including people living in Brazil, Ireland, New Zealand, and the United States [39–42]. The Canadian study by Hussain et al. [19] reported an increase in minor LEA rates from 2005–2016, which is interesting as Hussain defined minor LEA to include amputation at the level of the ankle whereas we identified ankle amputation as a major amputation [42].

Finally, our finding that 27.8% of all LA performed in Saskatchewan are subsequent LA, which includes both revision amputation and contralateral amputation, are concordant with the findings of other authors who report revision rate as high as 30% in LA due to PAD and 37% after traumatic LA [43, 44]. Our finding of a 9.6% increase in subsequent minor LA is supported by Dillingham et al. who report higher rates of reamputation after minor primary amputation in people with PAD [10]. The desire for limb preservation to enhance function as a possible precursor to revision amputation is well documented and may be the reason for the high rate of subsequent minor LA in our population [41, 42].

As a whole, the stable rate of LA over the 14-year study period was largely due to a decline in the rate of overall major LA concomitant with both a decline in the rate of primary major LA and a rise in the rate of subsequent minor LA rates. This rising rate of more frequent and repeated minor LA may reflect changing intervention strategies implemented to maintain limb function, especially in the presence of DM and PVD. The importance of long-term surveillance to understand rates of major and minor LA considering primary and subsequent intervention is an important step to evaluate and initiate prevention and limb loss management programs.

## Strengths and limitations

To the best of our knowledge, this is the first study to have explicitly explored trends of amputation rates, over a decade, in Saskatchewan. The current study used three diverse approaches, including two robust statistical methods to explore the LA trends. Further, the LA rates were calculated from equal time intervals of cases (in years) and population numbers (in years) hence making the rate more representative of annual provincial amputation rates. Our analysis was limited as APF were not adjusted for in the estimation of the relative rate and both overall LA or level of LA (minor or major) rates were not further disaggregated into cause-specific rates or location-specific rates within the broader categories of minor and major amputations (e.g., trans-femoral vs. trans-tibial).

## Conclusion

Our novel study examined all-cause amputation to quantify the actual burden of LA on the healthcare system, family, and caregivers. Overall LA rates remained stable over the study period with declining rates of major LA countered by rising rates of minor LA. Minor LA exceeded major LA with the largest rate increase identified in subsequent minor LA. Diabetes was the greatest APF for all LA groups. This rising rate of more frequent and repeated minor LA may reflect changing intervention strategies implemented to maintain limb function. The importance of long-term surveillance to understand rates of major and minor LA considering primary and subsequent intervention is an important step to evaluate and initiate prevention and limb loss management programs.

## Data Availability

The datasets used and/or analyzed during the current study are available from the corresponding author on reasonable request.

## Abbreviations

LA: Limb Amputation
SD: Standard Deviation
PAD: Peripheral Arterial Disease
DM: Diabetes Mellitus
PORT: Patient Oriented Research Team
DAD: Discharge Abstract Disease
CCI: Canadian Classification of Health Intervention
APC: Annual Percent Change
AAPC: Average Annual Percent Change
CI: Confidence Interval
ICD: International Classification of Disease
RR: Relative Rate
